# Therapeutical and Pharmaceutical Notes

**Published:** 1899-07

**Authors:** W. J. Martin

**Affiliations:** Kankakee, Ill.


### THERAPEUTICAL AND PHARMACEUTICAL NOTES.

UNDER THE DIRECTION OF W. J. MARTIN, M.D.C.,
KANKAKEE, ILL.

ABSORPTION AND EXCRETION OF IRON. A. Hoffmann's, Zurich (*Virchow's Archiv.*), micro-chemical investigation of the wall of the alimentary canal shows that in man the principal absorption is in the duodenum. The appearances are little different when medicinal doses of iron are added to the food; the liver, and especially the spleen, are places where the iron is stored, whilst it is excreted by the kidneys and large intestine. Analogous results were found in the guinea-pig. It is believed that inorganic salts of iron are capable of absorption.

POISONOUS PLANTS. It may interest veterinarians to know that the berries of the yew have killed many persons, and it is pretty well known nowadays that it is not safe to eat many peach-pits or cherry-kernels at once. Says the *Lancet*, New York: Among the garden-plants generally in vogue which possess a poisonous nature, botanists mention the jonquil, white hyacinth, and snowdrop; the narcissus being also particularly deadly—so much so, indeed, that to chew a small scrap of the bulbs may result fatally, while the juice of the leaves is an emetic. There is enough opium in red poppies to do mischief, and the autumn crocus, if the blossoms are chewed, cause illness. The lobelias are all dangerous; their juice, if swallowed, producing giddiness, with pain in the head. Lady-slipper poisons in the same way as does lobelia; the bulbs seem to be most harmful. Lilies of the valley are also poisonous. The leaves and flowers of the oleander are deadly, and the bark of the catalpa tree is very mischievous. The water-dupwort, when not in flower, resembles celery, and is virulent.

ERRORS IN X-RAY PHOTOGRAPHS. It has been recently pointed out by Tait (*Proceedings Cal. Academy of Med.*) that errors in radiography may lead to serious, if not fatal, results in cases where the x-ray apparatus has been employed in making examinations as an aid to diagnosis in medicine and surgery. Dr. Tait mentions several cases of inaccurate photographs of internal structures obtained by the x-ray method. He mentions one case in particular where a radiograph showed a lesion of the tenth dorsal vertebra, but another picture of the same part two weeks later failed to show any pathological change whatever, which was decidedly an error. Other errors of like nature have been reported by investigators: One of these mentions[1] a case where a radiograph showed a foreign body in the tissues, when upon operating none could be found.

As to what are the causes of these errors of x-ray photographs, scientists are as yet unable to decide; the time to which an object is exposed to the rays seems to be very important, as diametrically opposite pictures have been obtained of the same objects under different periods of exposure to the rays. The position from which the rays of the light originate, and the different positions from which patients and objects are placed before the Crooke's tubes of which radiographs are desired to be secured, also seem to be an important factor in securing accurate photographs.

From the above it would appear that where the best results are desired by this method, in securing a correct photograph of internal morbid pathological condition as an aid to diagnosis, it should be the aim of the medical practitioner to employ only a skilled operator, one who is entirely familiar with the many intricate problems which surround the x-ray apparatus. By doing so he will avoid the serious and perhaps fatal blunders that would most likely follow the efforts of an amateur operator.

TO RENDER CREASOTE SOLUBLE IN WATER. Saponin is said to have the property of rendering creasote soluble in water. To 10 grammes of beechwood creasote add 80 grammes of tincture of yuellaja and 60 grammes of distilled water. This mixture forms a solution which can be diluted with tepid water and administered as an enema or otherwise.—*Merck's Archives.*

CREOLIN AS A DEODORANT OF IODOFORM. Iodoform, it is said, may be completely deodorized by creolin. Vaczi recommends for that purpose, iodoform, 2 parts; creolin, 1 part; petrolatum, 25 parts.—*Ibid.*

DURING the last forty-one months the Chicago Health Department treated with antitoxin 4000 cases of true diphtheria, with a mortality of less than 6–8 per cent. In former years

[1] Druggists' Circular.

the deaths from diphtheria treated without antitoxin often reached as high as 35 per cent.

PHENOL PASTILLES. These are made by melting together 95 parts of pure phenol and 5 parts of curd soap, and pouring into cooled moulds. They keep well and are easily soluble in water.—*Can. Phar. Journal.*

MAGNESIUM HYDRATE AS AN ANTIDOTE IN POISONING BY ARSENIC. Dr. Glucksmann (*Zeischr. f. Allg. Oester. Apoth. ver*) has found that ferric hydrate is not a satisfactory antidote in that it does not completely precipitate the arsenic, but by using freshly prepared magnesium hydrate better results were obtained. The method which he recommends is the following: 50 grammes of magnesium sulphate is dissolved in 250 c.c. of distilled water, and to the solution is added 15 grammes of soda dissolved in 250 c.c. of water. The mixture may, in case of necessity, be given at once.

This mixture may also be given in cases of poisoning by alkaloidal salts, mercuric chloride, phosphorus, and in all cases where the nature of the poison is not know.—*Can. Pharm. Jour.*

ACTION OF BORIC ACID AND BORAX ON PEPSIN AND PANCREATIN. Dr. Keppler has investigated the effect of boric acid and borax on the digestive action of pepsin and pancreatin (*Pharm Centi.*, Jan. 12th), and finds that practically the same quantities of albumin are converted into albumose and peptone whether boric acid be present or not. The conversion of starch into invert sugar by means of pancreatin did not seem to be retarded by the presence of these preservative agents.—*Ibid.*

FOR A MORE ACCURATE DIAGNOSIS OF TUBERCULOSIS. The discussion on "pseudotuberculosis" at the Pathological Society of London drew a full house. Dr. Sims Woodhouse, in opening, commented on the unsatisfactory character of the term "pseudotuberculosis," which has been employed from time to time by different observers to cover such a variety of lesions. He referred to a series of cases which had been detailed in the report of the Royal Commission on tuberculosis, in which the naked-eye appearances must unquestionably have led to the diagnosis of tubercular disease, but for the negative evidence afforded by the microscope; these were associated with a variety of microörganisms quite distinct from tubercle bacillus. He had also seen small glistening pearly nodules produced by strongylus filariæ in the lungs of sheep included among the pseudotubercular lesions. The microscope showed that the nodules consisted of coils of nematoid worms surrounded by proliferating connective-tissue cells. Professor Muir, of Dundee, had described no less than six forms of pseudotuberculosis in birds. Then, again, in many museum specimens, it was clear that cases of actinomycosis had been gathered up into the category of pseudotubercular lesions. Professor Boyce had rescued from this appellation the mycotic condition of the lung due to aspergilli, which Kottjar had described as "aspergillar pseudotuberculosis," but which Boyce termed "aspergillo-pneumonomycosis." The confusion was still further increased by some observer describing a "pseudotuberculosis bacillus" resembling the tubercle bacillus in its morphology and staining reaction, but not associated with the occurrence of pseudotubercular lesions. The position was this: On the one hand they had organisms of pseudotuberculosis which had the morphological and staining characters of the true tubercle bacillus, but which pathologically appeared to be widely separated from it, whilst on the other hand they had a whole series of lesions which presented certain superficial resemblances to tubercle, but which were not induced by the action of the tubercle bacillus. It was very difficult to say what typical tubercle is, seeing that the forms of histological lesion associated with tubercle are so manifold. There was not sufficient evidence to justify the accepting of "pseudotuberculosis" as a pathological entity. It was well to be precise in the use of the term "tuberculosis," and to regard the presence of the tubercle bacillus as the essential factor; on the other hand, it would make the position much clearer if the term "pseudotuberculosis" were rejected altogether, and an effort made to refer to their proper place the essentially distinct lesions grouped under this inappropriate name.—*Therap. Gazette.*

EFFECTIVE PERMANGANATE GERMICIDE. Kronig and Paul recommend the following as a most effective germicide. Dissolve 20 grammes of potassium permanganate in 500 c.c. of water, and add to a mixture of 45 c.c. of hydrochloric acid in 1600 c.c. of water. Spots produced by this solution may be removed with 1–5 oxalic acid solution.

Incompatibilities of Tinctures. Tinctures being the products of the action of alcohol on vegetable principals, says M. Mausier (*Cent. Mech. et Pharm.*) would at first seem to be peculiarly adapted to mixtures of the most complicated description, yet, as an actual fact, the opposite is the result. Thus we see every day, tincture of calumba associated with tincture of kola, or cinchona, or canella, tinctures gorged with tannin, which at once precipitate the active principle of calumba (mene spermine) and leave the mixture almost inert, on account of the loss on the one side of astringent and on the other of active principles.

Value of Injection of Salt Solution. F. Macini (*Clinica Chir.*) says that besides the mechanical effect of the transfusion of normal salt solution in increasing the blood-pressure, and altering the pulse, respiration, and temperature, there is also a chemical effect which explains to some extent the constant elevation of temperature and the alteration in the blood, at first diminishing the number of blood-corpuscles and the amount of hæmoglobin, and later increasing them beyond the normal proportion.

Treatment of Carbolic Acid Poisoning. Hamsperger, (*Charlotte Med. Jour.; Med. News*) saw a boy, aged sixteen years, within thirty minutes of the time he had swallowed 1–5 oz. of carbolic acid. He was in a limp and comatose state, the pulse imperceptible. A pint of cream was at once poured into the stomach, which was kneaded in order to mix thoroughly the cream and the carbolic acid. Dry heat and friction were applied to the legs and arms. In two or three hours consciousness returned. The administration of cream and unskimmed milk was continued at short intervals for several hours. The patient entirely recovered in two days. Hansberger has found that an adult can take 4 drachms of pure carbolic acid mixed with cream and glycerin, or with alcohol, without any toxic symptoms developing.

Instability of Phosphorated Oil. Ekroor (*Arch. d. Pharm.*) has carefully investigated phosphorated oil, and arrived at the conclusion that this preparation should always be freshly prepared in order to obtain the best therapeutic results. He finds that a portion of the phosphorus combines with the oil, thus losing its elementary nature and that the percentage thus combined increases with the lapse of time.

